# When walking is bad for your back: a cohort study of risk factors for traumatic spinal injury in Abuja

**DOI:** 10.11604/pamj.2019.33.60.17565

**Published:** 2019-05-28

**Authors:** Benjamin Dean Holmes, Ruta Brazauskas, Emmanuel Adoyi Ameh, Oluwole Olayemi Olaomi, Laura Dawn Cassidy

**Affiliations:** 1 Institute for Health & Equity, Medical College of Wisconsin, Wisconsin, United States; 2 National Hospital Abuja, Abuja, Nigeria

**Keywords:** Road traffic injury, traumatic spinal injury, trauma registry, pedestrian, Nigeria, global health

## Abstract

**Introduction:**

This study evaluates characteristics and risk factors of traumatic spinal injuries (TSIs) treated at a trauma center in Abuja, Nigeria. TSIs are a global concern. They are frequently disabling, leading to economic, workforce, and quality of life strain. Little is known of the epidemiology of TSIs in Nigeria.

**Methods:**

Data were collected from National Hospital Abuja's trauma registry on 3025 patients treated at the hospital between 2014 and 2017. Patient characteristics were compared between spinal and nonspinal injury groups. Multiple logistic regression was used to identify risk factors of TSIs.

**Results:**

15% (452) of all injuries were spinal. Road traffic crashes were a significantly greater cause of spinal (77.4%) than nonspinal (59.4%) injuries (p<0.0001). Pedestrians were involved in 19% (356) of total crashes, occupying a significantly larger proportion of spinal (18.6%) than nonspinal (10.6%) injuries (p<0.0001). Three variables were modeled as risk factors of crash-related TSIs: mode of transportation, age, and gender. Only mode of transportation demonstrated statistical significance, with involvement as a pedestrian showing an adjusted odds ratio of 1.38 (95% confidence interval: 1.03-1.85, p=0.0329).

**Conclusion:**

Determining characteristics and risk factors of TSIs is an essential step in addressing this health concern in Nigeria. Crashes are a significant cause of TSIs, and a quarter of TSI patients involved in a crash are pedestrians. Involvement in a crash as a pedestrian is associated with high risk of TSI. These results can help guide both the development of spinal injury prevention policies and the allocation of resources.

## Introduction

Traumatic spinal injuries (TSIs) are a global public health concern [1]. TSIs include a variety of injuries which can affect the spinal cord, nerve roots, osseous structures, and/or soft tissues surrounding the spinal column [1, 2], and are caused by acute trauma to the neck or back [3]. There is a dearth of global TSI data [2], though some traumatic spinal cord injury (TSCI) data have been published. Between 250,000 and 500,000 people globally suffer a spinal cord injury each year [4]. Spinal injuries are particularly concerning in low- and middle-income countries (LMICs) [2], where incidence rates of TSCI can reach 29.7 per million people, compared to 13.1 per million in high-income countries [5]. Most TSIs globally are road traffic crash-related [2, 6-11], though falls are the leading cause of TSCIs in Turkey [12] and are becoming a larger cause of TSIs in both the United States [13] and Canada [14]. In Nigeria occupational hazard has also been implicated as a significant cause of TSIs [15]. Crashes are rapidly becoming one of the leading causes of injury and death worldwide. They are predicted to become the seventh leading cause of death globally by 2030 [16], and will also in part due to related TSIs become the fourth leading cause of disability-adjusted life year (DALY) loss by that year [17]. Nigeria, in particular, is acutely affected by road traffic crashes. The crash-related mortality rate in 2012 was 162 per 100,000 in Nigeria, which is 636% higher than the global average of 22 per 100,000 [18]. A Nigerian has one chance in nine of dying due to a crash, and one chance in three of being injured due to one in his/her lifetime [19]. Up to 4 million Nigerians are injured in crashes annually [20]. In addition, crashes are the leading cause of death in the world for those aged 15 to 29 years [16]. This is of particular concern in countries like Nigeria which suffer from skilled workforce depletion [21], as the economic productivity potential of this age group is high [22].

Mortality from TSIs is concerning; however the disability rate and long-term consequences are severe [2, 14, 23-25]. Just fewer than 18% of TSCIs were associated with mortality in one study of trauma patients over a 15-year period in Lagos, Nigeria [10]. A much greater percentage-nearly half of patients involved in TSIs-may experience chronic disabling symptomatology [26, 27]. Furthermore, pain-related disability is more common following trauma to the spine than to other body parts [28]. TSIs can therefore lead to extensive medical treatment, financial and workforce strain [11, 14], and loss of quality of life for the TSI patient [9, 25]. Little is known of the epidemiology of TSIs globally, and especially in Nigeria, [29] though reports suggest a consistent rise in cases [10]. The study presented here provides novel information on characteristics and risk factors of TSIs in Abuja, Nigeria. It was hypothesized that involvement in a road traffic crash as a pedestrian is a risk factor of TSI. This study's results will inform the “urgent need to improve the quantity and quality of [spinal injury] data collection” [4] and facilitate and guide efforts toward prevention and treatment of TSIs in Nigeria [9].

## Methods

**Data collection**: data were collected from a trauma registry of patients seen at the National Hospital Abuja in the Federal Capital Territory, Nigeria. The registry is HIPAA-compliant, and all data from the trauma registry were de-identified and did not contain any protected health information. Trauma registry data collection began in 2010 following its inception as a joint project between Ahmadu Bello University Teaching Hospital in Zaria, National Hospital Abuja, and the Medical College of Wisconsin [30]. Currently only data from National Hospital Abuja are reported. Data were obtained from injury victims presenting to the National Hospital Trauma Centre by an attending physician after stabilization. A medical record staff member then entered the data into the registry. The following measures are included in the Trauma registry: presentation date, patient demographics, mechanism of injury, location of incident, patient escorts, prehospital care received (including transfers from other hospitals), vital signs, specific exams (imaging, serology, urinalysis), body regions examined (head/neck, chest, abdominopelvic region, upper extremities, lower extremities, and back), diagnosis (head injury, spine injury/paralysis, trunk injury, limb injury, and burns), treatment, ward transfer, outcome, and follow-up plan. The registry documents patient outcomes as alive (satisfactory, leaving against medical advice, admitted to an intensive care unit, awaiting surgery, pending, follow-up appointment, and discharged) and deceased. This study was approved by the Institutional Review Boards at both National Hospital Abuja and Medical College of Wisconsin/Froedtert Hospital.

**Patient population**: patients included in this analysis were categorized into spinal injury and nonspinal injury groups based on information gathered from the registry. Also, since cervical spine injuries accompany severe craniocerebral injuries caused by road traffic crashes [31-33], crash patients who scored 3 to 8 on the Glasgow Coma Scale (GCS) in the registry were included as spinal injury patients. Of a total of 3025 patients, 452 (14.9%) were determined to be spinal injury patients. These were classified by injury mechanism (road traffic crash, assault, burn, and fall), injury type (cord/neurological, head, fracture, penetration, and soft-tissue), and affected spinal level (cervical, thoracic, and lumbar/sacral). Of 1879 total road traffic injuries, 350 (18.6%) were determined to be spinal. Road traffic crashes were subclassified by mode of transportation: pedestrian; motorcycle, tricycle, or bicycle; or vehicle with four or more wheels.

**Statistical analysis**: the following patient characteristics were summarized with descriptive statistics: age, gender, injury mechanism, spinal injury type, and spinal region affected. It was assumed that the first three listed characteristics were most likely, of all recorded measures in the trauma registry, to be associated with TSI. Age, as a continuous variable, was compared between spinal and nonspinal injury groups using Wilcoxon rank-sum test. Both gender and injury mechanisms (including modes of transportation), as categorical variables, were compared via χ^2^ test. Multiple logistic regression analysis was used to evaluate crash-related TSIs in relation to age, gender, and mode of transportation. Statistical significance was defined as p<0.05. SAS OnDemand for Academics software (SAS Institute, Cary, NC) was used to perform statistical analysis.

## Results

A total of 3025 trauma patients treated at the National Hospital Abuja between September 2014 and February 2017 were included in the analysis. Table 1 summarizes descriptive statistics of patient demographics and injuries. The median patient age was 30 years (interquartile range, IQR: 22 to 38); ages ranged from 0 to 92 years. Most patients were male (75.9%, n=2289), and the majority of patients were injured in a road traffic crash (60.4%, n=1879). Pedestrians were injured in 19% (n=356) of recorded crash incidents, and 12% (n=224) of crashes involved motorcycles, tricycles, or bicycles. Pedestrians were more likely to sustain TSI (23.6% sustained TSI) than vehicle-users, i.e, those involved in crashes on motorcycles, tricycles, and bicycles, or in vehicles with four or more wheels (17.5%) (p=0.0075). Pedestrians were also more likely to be male (80.9%) than vehicle-users (75.0%) (p=0.0179). Pedestrians sustaining TSI were also more likely to die (26.2%) than their vehicle-user counterparts (16.1%) (p=0.0303). Road traffic crashes were the leading cause of TSIs (77.4%, n=350), followed by assaults (13.3%, n=60) and falls (9.1%, n=41). The cervical spine was the most frequently injured spinal region (70.4% of cases, n=318), leading both lumbar/sacral (19.7%, n=89) and thoracic (9.3%, n=42) regions (Table 1).

**Table 1:** Patient demographic and injury characteristics

patient characteristics	patient population (n=3025)
**Age**	
Median, years (IQR)	30(22-38)
Gender	
Female, n (%)	727(24.1%)
Male, n (%)	2289(75.9%)
Not recorded	9
**Injury mechanism**	
Assault, n (%)	464(15.6%)
Burn, n (%)	167(5.6%)
Pedestrian in a traffic crash, n (%)	356(12.0%)
Motorcycle/tricycle/bicycle in a traffic crash, n (%)	224(7.5%)
≥Four-wheeled vehicle in a traffic crash, n (%)	1299(43.7%)
Fall, n (%)	464(15.6%)
Unknown	51
**Spinal injury (n=452)**	
Cord/root, n (% of spinal injuries)	65(14.4%)
Head, n (% of spinal injuries)	187(41.4%)
Fracture, n (% of spinal injuries)	9(2.0%)
Penetrating, n (% of spinal injuries)	56(12.4%)
Soft tissue, n (% of spinal injuries)	135(29.9%)
**Injured spinal region (n=452)**	
Cervical, n (%)	318(70.8%)
Thoracic, n (%)	42(9.4%)
Lumbosacral, n (%)	89(19.8%)
Unknown	3
**Glasgow coma scale score**	
Severe (score of 3-8), n (%)	211(7.3%)
Moderate (score of 9-12), n (%)	193(6.7%)
Mild (score of 13-15), n (%)	2490(86.0%)
Unknown	131

IQR, interquartile range

**Figure 1:**
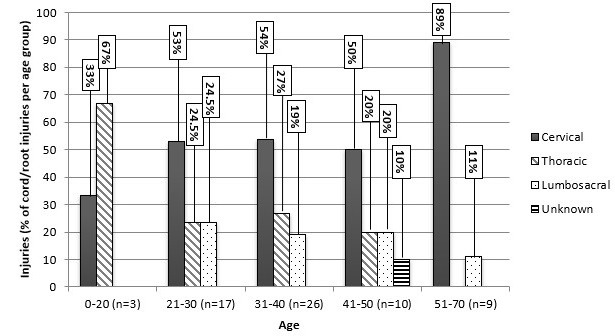
Traumatic spinal cord/nerve root injuries by age and spinal region

Patient discharge status and information on mortality were incomplete in 26% (798) of cases, and leaving against medical advice is reported in 9% (267) of general trauma cases, 6% (10) of crash cases, and 3.5% (16) of TSI cases. The trauma registry records 81 deaths in patients who sustained TSI, 73 of which were associated with severe craniocerebral injury. Spinal cord injury severity was measured in 15 of 57 cases: 5 of the 15 patients (33.3%) were classified as group A on the American Spinal Injury Association (ASIA) scale, meaning a complete loss of sensory and motor function in sacral segments S4-S5 [34]. One patient (6.7%) was classified as group B (sensory function is preserved, but motor function is not), three (20%) as group C (some motor function is preserved, with strength scored at 1-2 of 5), two (13.3%) as group D (some motor function is preserved, with strength scored at 3-5), and four (26.7%) as group E (normal sensory and motor function). Forty four percent (n=187) of all spinal injuries were accompanied by severe craniocerebral injuries (GCS score of 3-8), 30% (n=135) were primarily soft tissue related, and 14% (n=65) presented with spinal cord (n=57) or nerve root (n=8) pathology. Spinal injury type distributions were similar for road traffic crash patients. Of all spinal injuries, most affecting the spinal cord/nerve roots occurred in the cervical region for ages 21-70 but in the thoracic region for those aged 0-20 years (Figure 1).

Table 2 summarizes a comparison of patient demographic and injury characteristics by spinal injury. Age, gender, and four injury mechanisms (crashes involving pedestrians; crashes involving motorcycles, tricycles or bicycles; crashes involving vehicles with four or more wheels and falls) were each statistically significantly different between the two groups. The median age of spinal injury patients was two years greater than nonspinal injury patients, at 31 versus 29 years (p<0.0001). The percentage of males among those who sustained spinal injury (82% of TSI patients were male) was significantly greater than the percentage of males among the nonspinally injured (75% were male) (p=0.0021). Pedestrians and vehicle-users both occupied significantly larger proportions of TSI patients than of nonspinal patients, pedestrians more significantly so. Also, 43% (n=16) of patients aged 0-18 years who sustained spinal injury in a crash were pedestrians, compared to 15% (n=58) of patients older than 18 (p<.0001). Falls and burns were significantly more common injury mechanisms for nonspinal patients than TSI patients (p<0.0001 each).

**Table 2:** Relationship between injury type, age, gender, and injury mechanism

Patient characteristic	Spinal injury (n=452)	Nonspinal injury (n=2573)	P-value
**Age**			**<.0001**
Median, years (IQR)	31 (25-39)	29 (22-37)	
**Gender**			**0.0021**
Female, n (%)	83 (18.4%)	644 (25.1%)	
Male, n (%)	368(81.6%)	1921 (74.9%)	
**Injury mechanism**			
Assault, n (%)	60 (13.3%)	404 (15.7%)	0.2152
Pedestrian, n (% of crashes)	84 (18.6%)	272 (10.6%)	**<.0001**
Motor/tri/bicycle, n (% of crashes)	44 (9.7%)	180 (7.0%)	**0.0403**
≥Four-wheeled vehicle, n (% of crashes)	222 (49.1%)	1077 (41.9)	**0.0040**
Fall, n (%)	41 (9.1%)	423 (16.4%)	**<.0001**

IQR, interquartile range

Since road traffic crashes account for 60% of all injuries, focus was placed on identifying which crash patients are at highest risk of sustaining TSI. In order to explore that, univariate and multivariable logistic regression analyses were performed on three factors: mode of transportation, patient age, and patient gender, as summarized in Table 3. Two factors-mode of transportation and patient gender, showed statistical significance in univariate analysis (p=0.0077 and p=0.0155, respectively). In multivariable analysis only mode of transportation showed significance, demonstrating an association between TSI and involvement in a crash as a pedestrian (adjusted odds ratio, OR: 1.38, 95% confidence interval, CI: 1.03-1.85, p=0.0329) as compared to other modes of transportation (motorcycles, tricycles, or bicycles; and vehicles with four or more wheels). Multivariable analysis showed that patients 30 years old and older did not have a significantly higher risk of sustaining TSI than those younger than 30 (OR: 1.16, 95% CI: 0.91-1.49, p=0.2304) nor did male patients, compared to females (OR: 1.27, 95% CI: 0.95-1.71, p=0.1129) (Table 3).

**Table 3:** Logistic regression analysis of traumatic spinal injury

	univariate		multivariable	
Variable	Crude OR (95% CI)	P-value	Adjusted OR (95% CI)	P-value
Mode of transportation				
Vehicle	Referent		Referent	
Pedestrian	1.46 (1.11-1.93)	**0.0077**	1.38 (1.03-1.85)	**0.0329**
**Age, years**				
0-29	Referent		Referent	
≥30	1.16 (0.91-1.48)	0.2309	1.16 (0.91-1.49)	0.2304
**Gender**				
Female	Referent		Referent	
Male	1.43 (1.07-1.92)	**0.0155**	1.27 (0.95-1.71)	0.1129

OR, odds ratio

CI, confidence interval

## Discussion

This retrospective analysis described and compared characteristics of spinal to nonspinal injuries treated at the National Hospital Abuja Trauma Center from September 2014 to February 2017. It was hypothesized that being involved in a road traffic crash as a pedestrian is a risk factor of TSI. Nearly 15% of the 3025 injuries recorded in the trauma registry involved the spine, and 19% of the 1879 total road traffic crashes resulted in TSIs. While just over half of nonspinal injuries were caused by road traffic crashes, over three quarters of TSIs were caused by them. Given both the intensifying burden of road traffic crashes in Nigeria [35] and the high percentage of crashes which result in TSI, it is important to understand the mechanisms by which crash-related TSIs occur. One mechanism demonstrated in this review is pedestrian injury. Two of every 10 crashes involved pedestrians, and nearly a quarter of pedestrians involved in crashes sustained TSIs. TSI patients were significantly more likely to have been pedestrians than were nonspinal patients. Additionally, nearly half of children and adolescents who sustained spinal injury in a crash were pedestrians, compared to approximately one of every seven patients older than 18 years. Involvement in a road traffic crash as a pedestrian was associated with high risk of TSI. This greater risk of spinal injury for pedestrians involved in crashes could be explained by the increased vulnerability of the pedestrian's head/neck and back, as well as by a conceivably greater likelihood in a crash of direct impact to the pedestrian's spine. The greater likelihood of a child or adolescent, versus an adult, whose spine was injured in a crash to have been a pedestrian could be related to walking being the predominant mode of transportation for schoolchildren in sub-Saharan African cities [36]. Given the high risk of TSI for pedestrians and the fatal or debilitating effects of TSI, interventions for this vulnerable population should be explored. Such interventions could include: improving public awareness of the problem and of simple spinal dos and don'ts when encountering an injured pedestrian, expanding first responder training, and bolstering both human and structural resources for spinal health. Prevention measures should be pursued with equal vigor, including: improving driver behavior and decision-making, optimizing pedestrian visibility, with particular regard to the pediatric population and building safe mechanisms for transport to and from school.

This study has some limitations. First, it is a retrospective analysis and while the data were relatively complete upon admission, there were missing data throughout the course of the hospital stay and especially related to mortality. Second, it is informed solely by one hospital's trauma registry; however National Hospital Abuja is Federal Capital Territory's largest trauma center and has a wide catchment area. Third, because the Trauma Registry is new there is a lack of standardized injury severity assessment such as the abbreviate injury and ASIA scales. Finally, although involvement in a road traffic crash as a motorcycle, tricycle, or bicycle rider was not found to be significantly associated with TSI in this model, riders may be underrepresented in the trauma registry. Rapid urban growth in Nigeria over the past decade has sparked the burgeoning of transportation services, especially of intra-urban commercial motorcycle (or okada) services [37]. Although the Federal Capital Territory Administration banned okada use within Abuja city center in 2006, inconsistent enforcement has reportedly led to a thriving informal transportation sector including okadas [38], power bikes [39], and tricycles [40]. This legislation, while possibly of limited efficacy in decreasing okada use, has likely affected the reporting of okada-related crashes, injuries, and deaths however [41]. The mean ratio of motorcycle to total crashes in Federal Capital Territory between 2014 and 2016, as reported in Federal Road Safety Corps crash-scene data, was 14.2%, compared to 10.4% in the National Hospital Abuja Tauma Registry. Underrepresentation of motorcycle crash data in the Tauma Registry is possibly an upshot of riders' efforts to avoid formal medical assessment and potential legal citation, which could lead to vehicle confiscation [38, 42]. A greater likelihood than reported of TSI for motorcycle, tricycle, or bicycle riders involved in crashes is possible, due to the increased vulnerability both of the rider's head/neck (related to limited helmet use [43-45]) and of the back [46].

## Conclusion

Determining characteristics and risk factors of TSIs is an essential step in addressing this health concern in Nigeria. TSIs can assert a significant burden on both the individual patient and his/her community and economy. Road traffic crashes are a significant cause of TSIs in Abuja. A quarter of patients seen at National Hospital Abuja whose spines were injured in a crash were pedestrians, and nearly half of children and adolescents who sustained TSI in a crash were pedestrians. This study's findings suggest that a crash patient's status as pedestrian is a risk factor of spinal injury. The mechanisms undergirding these relationships warrant further research. Results from this and subsequent studies can help guide both the allocation of resources and the development of traffic-related TSI practice guidelines and interventional and preventative measures.

### What is known about this topic

- Most traumatic spinal injuries globally are road traffic crash-related;
- Road traffic crashes are predicted to become the seventh leading cause of death and fourth leading cause of disability-adjusted life year loss by 2030;
- The crash-related mortality rate in Nigeria is 162 per 100,000, which is 636% higher than the global average of 22 per 100,000.

### What this study adds

- Road traffic crashes are a significant cause of traumatic spinal injury in Abuja;
- Pedestrians were more likely than non-pedestrians to sustain a traffic-related traumatic spinal injury;
- A significantly higher percentage of children/adolescents who sustained spinal injury in a crash, compared to adults, were pedestrians.

## Competing interests

The authors declare no competing interests.
